# Differentiation associated regulation of microRNA expression *in vivo *in human CD8^+ ^T cell subsets

**DOI:** 10.1186/1479-5876-9-44

**Published:** 2011-04-20

**Authors:** Bruno Salaun, Takuya Yamamoto, Bassam Badran, Yasuko Tsunetsugu-Yokota, Antoine Roux, Lukas Baitsch, Redouane Rouas, Hussein Fayyad-Kazan, Petra Baumgaertner, Estelle Devevre, Anirudh Ramesh, Marion Braun, Daniel Speiser, Brigitte Autran, Philippe Martiat, Victor Appay, Pedro Romero

**Affiliations:** 1 Division of Clinical Onco-Immunology, Ludwig Center for Cancer Research of the University of Lausanne, Switzerland; 2 Department of Immunology, National Institute of Infectious Diseases, Shinjuku-ku, Tokyo, Japan; 3 Laboratory of Experimental Hematology, Bordet Institute, University of Brussels (ULB) Brussels, Belgium; 4 Department of Biochemistry, Laboratory of Immunology, Lebanese University, Faculty of Sciences, Hadath Beirut, Lebanon; 5 Infections and Immunity, INSERM U945, Avenir Group, Hôpital Pitié-Salpêtrière, Paris, France; 6 Department of Biological Sciences, Cornell University, Ithaca, NY

## Abstract

**Background:**

The differentiation of CD8^+ ^T lymphocytes following priming of naïve cells is central in the establishment of the adaptive immune response. Yet, the molecular events underlying this process are not fully understood. MicroRNAs have been recently shown to play a key role in the regulation of haematopoiesis in mouse, but their implication in peripheral lymphocyte differentiation in humans remains largely unknown.

**Methods:**

In order to explore the potential implication of microRNAs in CD8^+ ^T cell differentiation in humans, microRNA expression profiles were analysed using microarrays and quantitative PCR in several human CD8^+ ^T cell subsets defining the major steps of the T cell differentiation pathway.

**Results:**

We found expression of a limited set of microRNAs, including the miR-17~92 cluster. Moreover, we reveal the existence of differentiation-associated regulation of specific microRNAs. When compared to naive cells, miR-21 and miR-155 were indeed found upregulated upon differentiation to effector cells, while expression of the miR-17~92 cluster tended to concomitantly decrease.

**Conclusions:**

This study establishes for the first time in a large panel of individuals the existence of differentiation associated regulation of microRNA expression in human CD8^+ ^T lymphocytes *in vivo*, which is likely to impact on specific cellular functions.

## Background

CD8^+ ^T cells are major players of the immune response against viruses and cancers. Even though they represent a heterogeneous population, the expression of specific surface molecules characterizes distinct subsets (i.e. central memory, early, intermediate or late effector memory cells), which define the major steps of a process of memory T cell differentiation [1,2]. These multiple subsets present specific transcriptional programs, and therefore distinct range of receptors and intracellular proteins, indicating quite different requirements for stimulation and survival, homing potential and effector functions (reviewed in [3]). For instance, expression of effector molecules such as perforin or granzymes is restricted to the late stages of differentiation [4,5], while central memory cells are known for their superior proliferative capacity and often considered as the memory "precursors". However, the molecular mechanisms controlling peripheral CD8^+ ^T lymphocyte differentiation, and therefore the generation of immunological memory, remain poorly understood in humans.

MicroRNA (miRNA) are 18-22 nucleotide long RNA molecules that regulate gene expression at the post-transcriptional level through base pairing to partially complementary sites in the 3' UTR of the messanger RNA and integration into RNA induced silencing complexes (RISC) (reviewed in [6]). Inhibition of translation or degradation of the miRNA-bound mRNAs modulate protein output, inducing profound physiological effects. MicroRNA, which expression is tightly regulated during lymphopoiesis [7], recently emerged as key regulators of gene expression in the mammalian immune system (reviewed in [8,9]).

Although the biological functions of most miRNAs are not yet fully understood, unequivocal evidence for their role in lymphocyte development have been gathered in mouse models in recent years. For instance, the conditional deficiency in mouse lymphocytes of Dicer, the key enzyme in miRNA biogenesis, impaired lymphocytic differentiation [10,11]. Moreover, it was recently shown that abolishing Dicer expression in mature mouse CD8^+ ^T cells strongly impairs their response to pathogens *in vivo *[12], demonstrating that miRNAs are not only important for lymphocytes differentiation, but also for their functions in the periphery. Interestingly, single miRNA such as miR-181 have also been shown to be crucial in mouse haematopoiesis [13]. Thus, mouse models and/or studies on *in vitro *differentiated cells suggested a role for miRNAs in lymphocytes functions [14-16]. However, the modulation of their expression along antigen-driven differentiation of peripheral human CD8^+ ^T lymphocytes *in vivo *has not been studied yet.

We thus addressed this issue using microarrays and quantitative PCR to investigate microRNA expression profiles in different subsets of *ex vivo *sorted human CD8^+ ^T lymphocytes. Our results show the first evidence that these cells express a limited set of microRNAs, some of which displayed differential expression in differentiated CD8^+ ^T cells when compared to naive cells. The microRNA expression regulation uncovered here is likely to strongly influence the gene signature of these different subsets, and therefore to directly impact on their functional properties.

## Methods

### CD8 T cell subsets purification

Peripheral blood CD8^+ ^T lymphocytes were purified from healthy volunteers in agreement with local ethics committees (Cantonal Commission of Ethics in Research, State of Vaud, Switzerland, authorization #87/06) as described [4]. Briefly, total CD8^+ ^T cell preparations were obtained from leukapheresis by magnetic bead enrichment (Myltenyi Biotech, Bergish Gladbach, Germany) and stained with a combination of antibodies to CD3, CD8, CD45RA, CCR7, CD27 and CD28. All antibodies were from BD Pharmingen (San Diego, CA) except anti-CD45RA purchased from Beckman Coulter (Paris, France). The different subsets were then separated on a FACS Aria device (BD Bioscience, Allschwil, Switzerland) to routinely over 99% purity cell suspensions. Cells were either sorted directly to RNA lysis buffer (RNAlater, Qiagen) and frozen (for single specific qPCR assays) or into cold medium (for TaqMan Low Density Array experiments), before washings in ice-cold PBS and lysis (miRVana kit, Ambion).

### MicroRNA expression analysis

The TaqMan^® ^Low-density arrays (TLDA, Applied Biosystems, Foster City, CA) were used following manufacturer's instructions to simultaneously detect expression of 364 individual microRNAs. Briefly, total RNA was extracted with the miRVana kit (Ambion), quantified with a Nanodrop Spectrophotometer, and microRNA mature forms were reverse transcribed (30 min at 16°C, 30 min at 42°C, 5 min at 85°C) with Multiplex RT human primers and 100 mM dNTPs, 50 U MutliScribe reverse transcriptase, 20 U RNase inhibitor, 10 × RT buffer (8 reactions with pools of 48 stem-loop RT primers, 100 ng RNA per reaction). cDNA were then amplified on microfluidic cards in an ABI Prism 7900 HT with single microRNA specific primers (40 cycles of 15 s at 95°C and 1 min at 60°C). The same protocol was applied for single microRNA specific qRT-PCR (miR-17-3p, miR-17-5p, miR-19b, miR-20a, miR-92, miR-21, miR-155, miR-142-3p, miR-142-5p, and RNU44) from 10 ng total RNA, reverse transcribed with TaqMan Reverse transcription MicroRNA kit (Applied Biosystems) and amplified using Universal Fast Start Rox Probe Master Mix (Roche) and microRNA Assay kits in 384 well plates (Applied Biosystems) on a ABI Prism 7900 HT device (Applied Biosystems). Ct (RNU44) was subtracted to Ct (microRNA) to calculate relative expression (ΔCt).

### Statistical analysis

Statistical significance was evaluated with the GraphPad Prism software on non normalized expression data using the Friedman test (for non-parametric, paired data set) with Dunn's multiple comparison correction applied. Results were considered significant for p values < 0.05.

## Results and Discussion

### MicroRNA expression profiling in mature CD8^+ ^T lymphocyte subsets

Different subsets of human CD8^+ ^T lymphocytes can be defined based on the expression of CCR7, CD45RA and CD28 [4]. Naïve cells are CCR7^+ ^(and CD45RA^+ ^CD28^+^) while the effector memory subsets are CCR7^-^. Among the latter, the CD45RA^+ ^CD28^- ^cells representing the end stage of differentiation (late effector memory, L-EM), while CD45RA^- ^cells can be further divided in two functionally distinct subsets, with CD28^+ ^cells (early effector memory (E-EM) closer to central memory cells) being less differentiated than CD28^- ^subsets (I-EM), which share features with fully differentiated effectors (L-EM).

**Figure 1:**
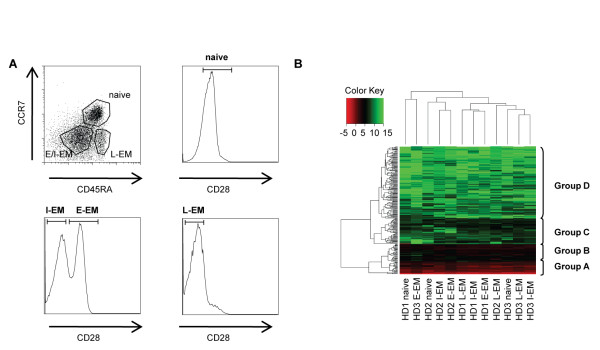
**Sorting of human CD8^+ ^T lymphocytes and microRNA large scale expression profiling**. a) CD8^+ ^T cells from healthy volunteers were FACS-sorted based on their expression of CD45RA, CCR7 and CD28 to isolate Naive (CD45RA^+ ^CCR7^+^, CD28^+^), E-EM (CD45RA^- ^CCR7^-^, CD28^+^), I-EM (CD45RA^- ^CCR7^-^, CD28^-^) and L-EM (CD45RA^+ ^CCR7^-^, CD28^-^). Data shown are gated on CD3^+ ^CD8^+ ^cells. b) Unsupervised clustering of the expression levels (ΔCt, normalized to RNU44) of 223 unique microRNAs (detected in at least one sample, i.e. Ct < 36) in CD8^+ ^T lymphocyte subsets from 3 healthy donors (HD1, HD2, HD3) as measured by TLDA (green: low expression/red: high expression).

These human CD8^+ ^T lymphocyte subsets (naive, E-EM, I-EM and L-EM, ordered from least to most differentiated) were purified from peripheral blood leukocytes from 3 healthy donors as indicated (Figure 1A), and the relative expression of 365 microRNAs was analyzed in these subpopulations using TaqMan Low Density Arrays (TLDA). Unsupervised clustering of relative expression levels in all subsets identified 4 main groups of microRNAs (Figure 1B): highly expressed (group A, 22 microRNAs, see Table 1), intermediate (group B, 31 microRNAs, see Table 1), low (group C, 46 microRNAs) and rare/absent (group D, 267 microRNAs). In total, 97 microRNAs were expressed well over detection limit, a figure similar to the 113 microRNAs recently detected in B cells using the same TLDA [17]. This list is likely not comprehensive, since the set of microRNAs detected with TLDA does not cover the whole range of microRNAs cloned in human. However, the low number of microRNAs considered expressed at high levels (group A) is in agreement with previous cloning studies in mouse CD8^+ ^lymphocytes [15,18] showing that a few highly expressed microRNAs prevailed in frequency. Interestingly, these microRNAs include miR16, miR-21, miR-142-3p and miR-142-5p, which were also found in the group A in human CD8^+ ^T lymphocytes, with miR-142-3p showing the highest relative expression levels (Table 1). MiR-26a/b and miR-146a/b also clustered to this group. In addition, 7 microRNAs of the 17~92 and paralog 106b~25 clusters (namely miR-19a, miR-19b, miR-20a, miR-25, miR-92, miR-93 and miR-106b) were identified among the 53 most expressed microRNAs (groups A and B, see Table 1). MiR-17-5p and miR-17-3p are expressed at lower levels (belong to group C), indicating intra-cluster differential expression.

**Table 1:** Human CD8^+ ^T cells express a limited set of microRNA

Group A		GROUP B	
**rank**	**miR**	**rank**	**miR**

**1**	hsa-miR-142-3p	**23**	hsa-miR-31

**2**	hsa-miR-26a	**24**	hsa-miR-125a

**3**	hsa-miR-16	**25**	hsa-miR-565

**4**	hsa-miR-26b	**26**	hsa-let-7b

**5**	hsa-miR-146b	**27**	hsa-let-7a

**6**	hsa-miR-29a	**28**	hsa-miR-181b

**7**	hsa-miR-19b *	**29**	hsa-miR-30e-3p

**8**	hsa-miR-92 *	**30**	hsa-miR-423

**9**	hsa-miR-342	**31**	hsa-miR-328

**10**	hsa-miR-30c	**32**	hsa-miR-101

**11**	hsa-miR-20a *	**33**	hsa-miR-223

**12**	hsa-let-7g	**34**	hsa-miR-27a

**13**	hsa-miR-142-5p	**35**	hsa-miR-25 *

**14**	hsa-miR-146a	**36**	hsa-miR-195

**15**	hsa-miR-24	**37**	hsa-miR-425-5p

**16**	hsa-miR-21	**38**	hsa-miR-222

**17**	hsa-miR-29c	**39**	hsa-miR-197

**18**	hsa-miR-30b	**40**	hsa-miR-28

**19**	hsa-miR-30a-5p	**41**	hsa-miR-186

**20**	hsa-miR-191	**42**	hsa-miR-331

**21**	hsa-miR-484	**43**	hsa-miR-192

**22**	hsa-miR-140	**44**	hsa-miR-103

		**45**	hsa-miR-594

		**46**	hsa-miR-15b

		**47**	hsa-miR-30e-3p

		**48**	hsa-miR-155

		**49**	hsa-miR-374

		**50**	hsa-miR-93 *

		**51**	hsa-miR-19a *

		**52**	hsa-miR-106b *

		**53**	hsa-miR-30d

MicroRNA ranking according to relative expression in CD8^+ ^T cells. Groups A and B are as identified by unsupervised clustering in Figure 1b. *: microRNA belonging to the miR-17~92 cluster.

Transgenic overexpression of miR-17~92 cluster in mouse lymphocytes was shown to induce lymphoproliferative disease [16]. High expression levels are therefore likely to be tightly controlled. The high expression levels found for a large set of microRNAs from the 17~92 cluster in primary human CD8^+ ^T lymphocytes had not been reported yet, and strongly suggests a role for this set of microRNAs in the biology of CD8^+ ^T cells.

### MiR-21 and miR-155 are upregulated during CD8^+ ^T lymphocyte differentiation

**Figure 2:**
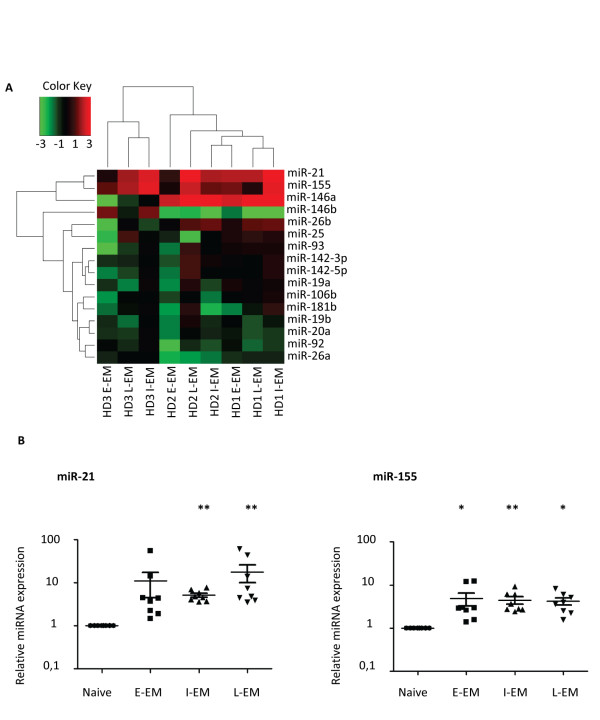
**miR-21 and miR-155 are upregulated in differentiated CD8^+ ^lymphocyte subsets**. a) Unsupervised heatmap clustering of the expression levels of a selected set of microRNAs in each subset normalized to levels in naive cells (log2 fold change) (green: downregulated/red: upregulated). b) Expression levels of miR-21 and miR-155 in sorted human CD8^+ ^T cell subsets were determined by single specific qPCR performed in triplicates, and are expressed as fold change relatively to levels in naive cells (n = 8; *: p < 0,05; **: p < 0,01).

To investigate if defined microRNA profiles can be associated with CD8^+ ^T cell differentiation status, the relative expression levels of the microRNAs from groups A, B and C were calculated for each CD8^+ ^lymphocyte subset relative to those found in the naive cells. Global unsupervised clustering did not identify clear microRNA clusters, and grouped subpopulations from the same donor together, due to strong inter-donor heterogeneity. However, specific patterns could be highlighted when the analysis was focused on a more restricted set of microRNAs (Figure 2A), chosen for their suggested role in immunity. The well expressed miR-21, miR-155 and miR-146a clustered together as consistently upregulated, while the abundant microRNAs of the miR17~92 clusters (miR-19b, miR-20a and miR-92) showed a clear trend towards decreased expression in differentiated cells, as did miR-26a (Figure 2A).

MiR-21 has been shown to be upregulated in mouse CD8^+ ^T cells upon *in vitro *activation [15], but its function in these cells remains unknown. MiR-155, encoded by the BIC transcript, is induced upon antigen-receptor triggering in lymphocytes and was shown to be instrumental for the generation of adaptive immunity in mouse [19,20]. Our TLDA analysis suggested that both miR-21 and miR-155 were more expressed in *in vivo *differentiated human primary CD8^+ ^T cells than in their naive counterparts (Figure 2A). These results were confirmed by single specific RT-qPCR on eight additional donors, which all clearly showed higher expression of both miR-21 and miR-155 in antigen-experienced cells (Figure 2B).

Altogether these results demonstrate that an increase of miR-21 and miR-155 expression occurs upon *in vivo *differentiation of human CD8^+ ^T lymphocytes. Dynamic regulation of these microRNAs in mouse CD8^+ ^T cells has been described upon *in vitro *activation [15]. By contrast, the data presented here on *ex vivo *sorted lymphocytes revealed for the first time modulations that occurred during *in vivo *differentiation of human peripheral CD8^+ ^T cell subsets.

### The miR-17~92 cluster tends to be downregulated during CD8^+ ^T cell differentiation

**Figure 3:**
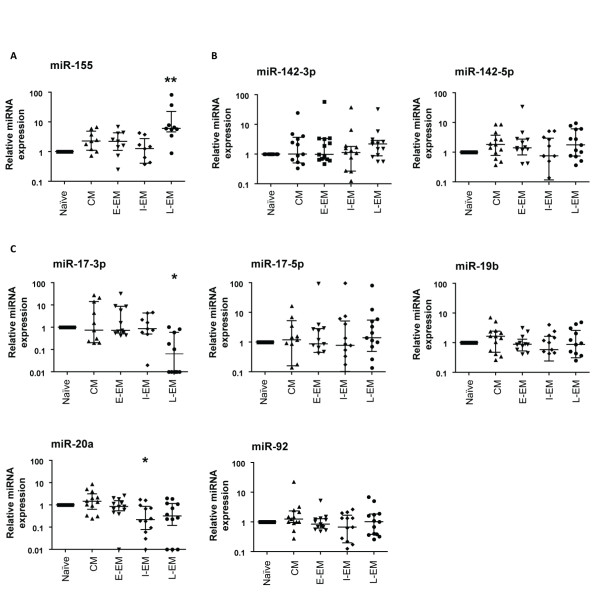
**The miR-17~92 cluster is preferentially downregulated in differentiated CD8^+ ^T cell subsets**. Expression levels of miR-155 (a), miR-142.3p and miR-142-5p (b) and of members of the miR-17~92 cluster (c) were determined by single specific qPCR in the indicated sorted human CD8^+ ^T cell subsets (a), and are expressed as fold change relatively to levels in naive cells (n = 9; *: p < 0,05; **: p < 0,01).

Since central memory (CM) CD8^+ ^T cells are present at extremely low frequency in peripheral blood, a new sorting strategy was then designed to include this subset in our analysis. Addition of anti-CD27 to the panel of cell surface molecule specific antibodies allowed the isolation of naïve (CCR7^+ ^CD45RA^+ ^CD27^+ ^CD28^+^), CM (CCR7^+ ^CD45RA^- ^CD27^+ ^CD28^+^), E-EM (CCR7^- ^CD45RA^+/- ^CD27^+ ^CD28^+^), I-EM (CCR7^- ^CD45RA^+/- ^CD27^+ ^CD28^-^) and L-EM (CCR7^- ^CD45RA^+/- ^CD27^- ^CD28^-^) subsets. Consistent with our previous analysis (Figure 2B), upregulation of miR-155 in antigen experienced cells versus their naïve counterparts was confirmed in the new set of 9 independent donors, with a clear trend toward highest expression levels in the most differentiated CD8^+ ^T cells (L-EM) (Figure 3A). Since the TLDA analysis showed that both miR-142-3p and miR-142-5p were among the most expressed microRNAs in CD8^+ ^T cells (Table 1), their expression levels were assessed in the different subsets. No significant regulation of expression could be found, likely suggesting a constitutive role rather than a differentiation induced function for these microRNAs (Figure 3B).

Seven members of the 17~92 cluster, which recently were shown to be critical regulators of lymphocyte development and proliferation [16], were identified among the 53 most expressed microRNAs in human CD8^+ ^T cells (Table 1), with a trend toward lower expression in more differentiated cells (Figure 2A). Expression levels of miR-17-3p, miR-17-5p, miR-19b, miR-20a and miR-92 were therefore determined by single specific qPCR in differentiated CD8^+ ^T cell subsets, and compared to the levels found in naïve cells. In agreement with the TLDA data, and despite a relatively important inter-donor variability, the expression of some of these miRNAs was found to be preferentially associated with specific subsets of CD8^+ ^T cell differentiation. Interestingly, the expression level of miR-20a was significantly increased in the central memory subset, in contrast to more differentiated subsets. Along the same lines, miR-17-3p expression was significantly decreased in late effector memory cells. There were also non significant trends towards preferential expression of miR-19b and miR-92 in the central memory cells. MiR-17-5p expression showed no association with CD8^+ ^T cell differentiation. On the whole, expression of several members of the miR-17~92 cluster appeared to be found preferentially during early memory differentiation. Interestingly, not all members are regulated concomitantly, indicating a differential intra-cluster regulation in agreement with the TLDA results (Table 1).

Altogether, the data presented here demonstrate for the first time that human CD8^+ ^T cell subsets express a limited set of microRNAs, which include several members of the 17~92 and related clusters. Moreover, this study shows that the *in vivo *differentiation from naive cells is associated with a stage-specific regulation of distinct microRNA expression, with miR-21 and miR-155 being upregulated in antigen-experienced subsets, the more differentiated subsets expressing higher levels of these microRNA (Figures 2B and 3A). MiR-155 has been suggested to be part of a negative feedback loop downstream the Toll-like receptor/Interleukin 1 receptor pathway [21]. The results presented here may suggest similar functions in human CD8^+ ^T cells downstream of the TCR. Moreover, miR-155 has been recently shown to target SOCS-1 expression, both in tumor cells [22] and in mouse CD4^+ ^regulatory T cells, in which it modulates the cellular responses to IL-2. Whether miR-155 plays a similar role in CD8^+ ^T cells remains an interesting issue to investigate, as it might shed new light on the relationship between cytokine signaling and lymphocytes differentiation.

In contrast to the clear upregulation observed for miR-155, the expression of the miR-17~92 cluster (especially miR-17-3p and miR-20a) tended to decrease along differentiation. Our results are in line with a recent report by Hackl and colleagues [23] that shows downregulation of this cluster in CD28 negative CD8+ T cells. Considering the role of the miR-19~92 cluster in lymphocyte development and proliferation, it is tempting to speculate that its expression may be related to the greater proliferative potential and memory precursor-like capacity, characteristic of central memory cells compared to more differentiated subsets (in particular late effector memory cells). Interestingly, expression of this cluster is induced by the transcription factor c-Myc [24], which has been shown in the mouse to be a component of the IL-15-dependent pathway controlling homeostatic proliferation of memory CD8^+ ^T cells [25]. In addition, the 17~92 cluster has been experimentally shown to inhibit Bim expression [16], which plays a key role in activation induced cell death in lymphocytes [26]. Whether 17~92 induced regulation of Bim plays a function in CD8^+ ^T cells survival potential (greater in less differentiated cells) is an interesting issue to be further investigated.

## Conclusions

Mouse models have shown that the limited set of microRNAs expressed in mature CD8^+ ^T cells critically support their functions in response to pathogens [12,15]. Similarly, the differential microRNA expression profile observed here for the first time in human CD8^+ ^T cell subsets is likely to have functional relevance in lymphocyte biology, in view of proteomic experiments demonstrating that a single miRNA can directly repress the production of hundreds of proteins in mammalian cells, through both downregulation of mRNA levels and translation inhibition [27]. Further investigation is warranted to dissect the precise role of individual differentially expressed microRNA in the regulation of CD8^+ ^T cell functions. We are currently pursuing analyses on human CD8+ T cell clones and in mouse models to better understand the functional relevance of the results presented here. Understanding this novel aspect of lymphocyte biology will help to better define CD8^+ ^T cell differentiation, and might shed light on the relationship between differentiation and functional properties. In that respect, it might therefore be helpful to elaborate better vaccination strategies for induction of CD8^+ ^T cells with appropriate differentiation and functions.

## Competing interests

The authors declare that they have no competing interests.

## Authors' contributions

BS, TY, PM, YTY BA, VA, PR designed the study. BS, TY, BB, RR, HFK, PB, ED, AR, MB generated the data. BS, TY, BB, LB, YTY, DS, VA, PR analyzed the data. BS, VA, PR wrote the paper. All authors have read and approved the final manuscript.
